# Hospitalization Rates for Coronary Heart Disease in Relation to Residence Near Areas Contaminated with Persistent Organic Pollutants and Other Pollutants

**DOI:** 10.1289/ehp.7595

**Published:** 2005-03-14

**Authors:** Alexander V. Sergeev, David O. Carpenter

**Affiliations:** Institute for Health and the Environment, University at Albany, Rensselaer, New York, USA

**Keywords:** acute myocardial infarction, hazardous waste sites, hospitalization, persistent organic pollutants, Superfund sites

## Abstract

Exposure to environmental pollutants may contribute to the development of coronary heart disease (CHD). We determined the ZIP codes containing or abutting each of the approximately 900 hazardous waste sites in New York and identified the major contaminants in each. Three categories of ZIP codes were then distinguished: those containing or abutting sites contaminated with persistent organic pollutants (POPs), those containing only other types of wastes (“other waste”), and those not containing any identified hazardous waste site (“clean”). Effects of residence in each of these ZIP codes on CHD and acute myocardial infarction (AMI) hospital discharge rates were assessed with a negative binomial model, adjusting for age, sex, race, income, and health insurance coverage. Patients living in ZIP codes contaminated with POPs had a statistically significant 15.0% elevation in CHD hospital discharge rates and a 20.0% elevation in AMI discharge rates compared with clean ZIP codes. In neither of the comparisons were rates in other-waste sites significantly greater than in clean sites. In a subset of POP ZIP codes along the Hudson River, where average income is higher and there is less smoking, better diet, and more exercise, the rate of hospitalization for CHD was 35.8% greater and for AMI 39.1% greater than in clean sites. Although the cross-sectional design of the study prevents definite conclusions on causal inference, the results indirectly support the hypothesis that living near a POP-contaminated site constitutes a risk of exposure and of development of CHD and AMI.

Coronary heart disease (CHD) is the most common, serious, chronic, life-threatening illness in the United States, affecting more than 11 million people (Selwyn and Braunwald 2001). The major risk factors for CHD (smoking, hypertension, dyslipidemia, diabetes mellitus, male sex, and older age) are well known. However, environmental exposures may also contribute to the risk of development of CHD. Air pollution with particulate matter results in an elevated risk for cardiovascular mortality and an increase in hospitalization rates for cardiovascular diseases (Brook et al. 2004). In the Kuopio Ischemic Heart Disease Risk Factor Study, Salonen et al. (1991, 1992) found evidence that copper and iron are risk factors for heart disease and later demonstrated that higher dietary intake of mercury from non-fatty freshwater fish was associated not only with an increased risk of acute myocardial infarction (AMI) but also with CHD mortality, cardiovascular disease mortality, and any cause mortality among eastern Finnish men (Salonen et al. 1995); the researchers suggested that the mechanism occurs through promotion of lipid peroxidation. Guallar et al. (2002) also found a direct association between toenail mercury concentration and risk of myocardial infarction. Arsenic exposure is associated with peripheral vascular disease (Tseng et al. 1996). Occupational exposure to dioxins/furans and polychlorinated biphenyls (PCBs) has been correlated with an excess incidence of CHD (Calvert et al. 1998; Flesch-Janys et al. 1995; Gustavsson and Hogstedt 1997; Hay and Tarrel 1997; Vena et al. 1998).

Several reports have indicated elevations in rates of various diseases (Goldberg et al. 1995; Hall et al. 1996) and birth defects (Croen et al. 1997; Geschwind et al. 1992) among individuals living near hazardous waste sites. New York has a large number of relatively well-characterized hazardous waste sites, known by location and by the major contaminants present at each site. New York also has an excellent statewide system of reporting diseases diagnosed in every hospitalized patient in state-regulated hospitals, which was available to us on the basis of the ZIP code of residence of each patient for the years 1993–2000. We have matched these data sets to investigate the frequency of hospitalization diagnosis for various forms of CHD with residence in ZIP codes that either contain, or do not contain, hazardous waste sites with persistent organic pollutants (POPs) or other wastes.

Fat-soluble POPs, such as PCBs, dioxins/furans, and persistent pesticides such as dichlorodiphenyltrichloroethane (DDT) are of special concern because of their persistence in both the human body and the environment. This class of compounds bioconcentrate in food and can be ingested, inhaled, or absorbed through the skin. These compounds alter normal functioning of the immune, nervous, and endocrine systems and are carcinogenic (Carpenter 1998; Lucier 1991; Vine et al. 2000). In previous studies, we demonstrated elevated hospitalization for thyroid diseases, diseases of the genital tract, and endometriosis among hospitalized women residing within 15 miles of three areas of concern (AOCs) in western New York (Carpenter et al. 2001) and when comparing all ZIP codes containing or abutting POP-contaminated sites with either “other waste” ZIP codes (ZIP codes containing a hazardous waste site but not one containing POPs) or “clean” ZIP codes (ZIP codes that do not contain an identified hazardous waste site) (Carpenter et al. 2003). Kudyakov et al. (2004) reported a similar elevation in risk of infectious respiratory diseases and chronic bronchitis in POP-contaminated ZIP codes. Residential proximity to PCB-contaminated waste sites is associated with increased risk of giving birth to a low-birth-weight infant, especially if it is a male child (Baibergenova et al. 2003).

There is evidence in animals that PCBs induce lipogenic enzymes (Boll et al. 1998), that dioxin-like PCBs cause direct damage to endothelial cells (Toborek et al. 1995), and that workers occupationally exposed to dioxins and PCBs have elevated serum lipids (Calvert et al. 1996; Stehr-Green et al. 1986). Because elevated serum lipids are a major risk factor for CHD, we hypothesized that residency in proximity to POP sites may be associated with increased use of CHD hospital care services. The objective of our study was to examine the potential effect of residential exposure to POPs and non-POP contaminants on hospitalization rates for patients with AMI and other forms of CHD.

## Materials and Methods

We conducted a cross-sectional study of hospital discharge rates. Data on hospital discharges were obtained from the New York Statewide Planning and Research Cooperative System (SPARCS; New York State Department of Health, Albany, NY), which is an administrative database. Like other administrative databases, it contains formalized information derived/abstracted from data sets such as clinical charts. We used SPARCS data from 1993–2000, with some 2.5 million hospital discharge records collected annually. Up to 15 diagnoses and 15 procedures, coded according to the *International Classification of Diseases*, *9th Revision* (ICD-9; 1992), are recorded for every patient discharged from any hospital in New York State, except for federal hospitals. The SPARCS data set does not include data from New York City, which maintains its own hospitalization data. For this reason and because New York City differs from the rest of the state in many other ways, we excluded New York City from this study. Data were collected on all the hospital discharges that contained any of the ICD-9 codes for CHD [410.0–414.9 (ischemic heart disease) and 429.7 (sequelae of myocardial infarction, not elsewhere classified)]. The primary outcome variable was the CHD hospital discharge rate calculated as a number of hospital discharges of patients with a diagnosis of CHD divided by the total population residing in a given area (ZIP code). We analyzed separately data for ICD-9 code 410 (AMI), because AMI is more likely to be a primary diagnosis and is less likely to recur in a single individual.

Every SPARCS record contains information on patient’s age, sex, race, source of payment (including the type of health insurance coverage), and the ZIP code of their residence. We used this information to adjust for potential confounders. Income was employed as a proxy measure of socioeconomic status (SES), which is another potential confounder in our study. Data on income (median household income on the ZIP code level) were obtained from the 2000 U.S. Census (U.S. Census Bureau 2000).

**Figure 1:**
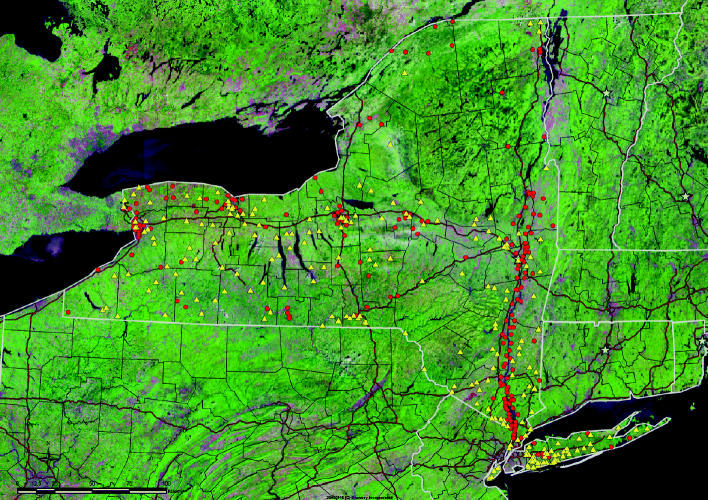
Map of New York State showing the locations of POP sites in red and other-waste sites in yellow. Map prepared by Rick Crowsey, Crowsey Incorporated.

The ZIP codes were classified as POPs, other waste, or clean, depending on whether they contained or abutted contaminated sites, where POPs were considered to be PCBs, dioxins/furans, or chlorinated and persistent pesticides. We considered the hazardous waste sites in New York identified by the U.S. Environmental Protection Agency (EPA) (87 National Priority List sites) (U.S. EPA 2003), the New York State Department of Environmental Conservation (NYSDEC) (865 state Superfund sites) (NYSDEC 2004b), and the International Joint Commission (six AOCs) (U.S. EPA 2004), and for each site identified the ZIP code(s) containing or abutting the site and the contaminants listed as being those of primary concern by these agencies. Detailed information on the federal and state Superfund sites, including amounts of contaminants they contain, insofar as that is known, is available on the U.S. EPA website (U.S. EPA 2005β); for the National Priorities List sites on the NYSDEC website (NYSDEC 2004a), and for the AOCs from a different U.S. EPA website (U.S. EPA 2004). Exclusive of New York City, there were 196 POP ZIP codes, 222 ZIP codes with other-waste sites, and 996 clean ZIP codes. Figure 1 is a map of New York State in which the ZIP codes with POP sites are shown in red, and those with other waste are shown in yellow. We analyzed separately the 78 ZIP codes in the PCB-contaminated portion of the Hudson River (200 miles from Hudson Falls to Manhattan), a National Priorities List site, because we have previously reported, using data from the Behavioral Risk Factor Surveillance System (BRFSS), that in those counties abutting the contaminated portion of the Hudson there is less smoking, more consumption of fruit and vegetables, and more frequent exercise than in the rest of the state (Kudyakov et al. 2004, their table 3). In addition, the average family income is greater in the ZIP codes along the Hudson River (Kudyakov et al. 2004, their table 2). Thus analysis of this subset of POP sites allows for partial control over the other major risk factors for CHD and AMI. In addition, we attempted to control for the adverse cardiovascular effects of particulate air pollution by use of the reported mean daily levels of 2.5 μm particulates as reported by the NYSDEC from its network of 43 air monitoring stations, with data reported on the NYSDEC website (NYSDEC 2004b) and Environmental Defense (2004). Effects of living in contaminated sites on the CHD and AMI hospital discharge rates were assessed using a negative binomial model. All analyses were performed using SAS statistical software (version 8.2; SAS Institute Inc., Cary, NC). GENMOD procedure in SAS was used to perform negative binomial regression.

## Results

Table 1 shows the demographics of the populations in each of the three categories of ZIP code for the period 1993–2000. Living in a ZIP code containing or abutting a POP hazardous waste site was associated with a 15% increased frequency of discharge diagnosis of CHD compared with living in a clean ZIP code (Table 2). Living in a ZIP code with other waste was also associated with a 4% elevated CHD diagnosis, but this was not statistically significant. As expected, CHD discharge rates were higher in men than in women and increased with age. Compared with CHD patients with median household income of < $30,312, those with higher income had lower hospital discharge rates. Coverage by Medicare (the U.S. government program for health care for the elderly) or comprehensive private health insurance, such as Blue Cross, was associated with lower use of in-patient hospital care services compared with Medicaid coverage (the U.S. government program for health care for the poor) or absence of insurance (self-pay). Hospitalization rates differed across racial/ethnic groups. Asians and Pacific Islanders had lower rates than did Caucasians, African Americans, and Native Americans.

Chronic forms of CHD would be expected to be coded often in the SPARCS database as one of the 14 “other diagnoses” (along with other comorbidities) rather than as the “principal diagnosis.” This may result in a bias problem. Nonsevere comorbidities that do not require treatment during the hospital stay may be undercoded, resulting in underestimation of the association between CHD hospitalization rates and exposure (bias toward null). But the relatively high prevalence of CHD in the general population makes it quite a common comorbidity. Higher hospitalization rates for any disease caused by the contaminants of interest would result in higher CHD prevalence among hospitalized patients, resulting in overestimation of the association between pollution and hospital care use by CHD patients.

To control for these possible biases, we analyzed the association between the most severe form of CHD (AMI) and ZIP code of residence. AMI is a very serious and often life-threatening disease, and it is less likely to be a comorbidity. Table 3 shows hospital discharge rates for AMI in relation to ZIP code of residence and adjusting for known covariates with negative binomial regression. After adjustment for the confounders, those residing in the areas contaminated with POPs have a significantly greater number of AMI hospitalizations. Residency in a POP-contaminated ZIP code is associated with a 20.0% increase in AMI hospital discharge rates compared with clean sites. The 7% elevation in AMI hospitalization rate in other-waste ZIP codes was not statistically significant.

Although other factors associated with AMI hospital discharge rates were not of primary interest for this study, they can be used for quality control purposes and thus merit careful examination. Male sex and older age are well-known risk factors for AMI and other forms of CHD, and more frequent hospitalizations should be expected in these population groups. Consequently, male sex and older age should be associated with higher hospital discharge rates for AMI. So adequacy of the model describing association between any exposure and AMI hospital discharge rates can be questioned if the model fails to indicate the contribution of sex and age.

The hospital discharge rate for AMI among males was about twice that among females, and it increased with age (Table 3). Compared with the lowest income category, those with higher household income had lower hospital discharge rates. Inequality in health insurance coverage is associated with some difference in hospital care use. Those covered by Medicaid or not insured (self-pay) had lower hospital discharge rates compared with those covered by comprehensive health insurance or by Medicare. Caucasians, African Americans, and Native Americans have higher discharge rates than Asians and Pacific Islanders. These results indirectly support the plausibility of the model.

There are other important risk factors for CHD, especially smoking, diet, and exercise. Information at an individual level for these risk factors is not available in our data sets. However, by use of BRFSS, we have county-level information, as reported previously (Kudyakov et al. 2004). In the counties along the 200 miles of the Hudson River that are contaminated with PCBs, there is less smoking, more frequent exercise, and more consumption of fruits and vegetables than in the rest of the upstate region. Tables 4 and 5 show the rates of hospitalization of residents in the 78 ZIP codes that abut the contaminated portion of the Hudson River for CHD and AMI, respectively. Despite living a healthier life style, residents along the Hudson River were 35.8% more likely to be discharged with a diagnosis of CHD, and 39.1% more likely to be discharged with a diagnosis of AMI. These results are highly significant.

Particulate air pollution is well documented to be an important risk factor for CHD and AMI. It is difficult to control for local differences in air pollution in an ecologic study such as this, but we have used what information is available from the air monitoring stations operated by the NYSDEC. Of the 43 stations in New York, 20 are outside of New York City, but in only 16 is regular monitoring of 2.5-μm particulates obtained (six in POP ZIP codes, seven in clean ZIP codes, and three in other-waste ZIP codes). The mean 24-hr 2.5-μm particulate levels reported were 11.5 μg/m^3^ (range, 7.7–13.7) in the POP ZIP codes, 11.1 μg/m^3^ (range, 10.5–12.2) in the other-waste ZIP codes, and 11.2 μg/m^3^ (range, 9.5–12.4) in the clean ZIP codes. Although the number of ZIP codes for which mean particulate information is available is small, the information that can be obtained does not suggest that air pollution is a major confounder.

## Discussion

The results of this study are consistent with the hypothesis that exposure to certain environmental contaminants increases the risk of development of CHD and AMI. Those persons residing in POP-contaminated ZIP codes have significantly higher rates of diagnosis of CHD and/or AMI on hospital discharge than do those living in noncontaminated areas. Residency in areas contaminated with other waste is also associated with an elevation in hospital discharge rates, but this relationship did not reach the traditionally used significance level of α = 0.05.

Others have reported health effects of living near hazardous waste sites [reviewed by Vrijheid (2000)]. Health Canada demonstrated a statistically significant elevation, relative to the rest of Ontario, in standardized morbidity ratios for a number of different diseases, including reproductive dysfunction, respiratory and gastrointestinal disease, and diabetes, in Canadian AOCs, highly contaminated sites along the Great Lakes as defined by the International Joint Commission (Elliott SJ et al. 2001). Congenital malformations have been found more commonly in residents living near hazardous waste sites in a number of studies (Croen et al. 1997; Dolk et al. 1998; Geschwind et al. 1992; Orr et al. 2002), whereas others have reported elevated incidence of low birth weight (Elliott P et al. 2001) and end-stage renal disease (Hall et al. 1996). Low birth weight is important in two regards: It is a known risk factor for the development of CHD later in life (Barker et al. 2002), and it has been demonstrated to occur more frequently in infants born to women occupationally exposed to PCBs (Taylor et al. 1989). Using New York birth registry data, we have previously demonstrated an elevation in rates of low-birth-weight infants (especially males) in residents of PCB ZIP codes after adjustment for other factors, including mother’s age, race, weight, height, and incidence of smoking (Baibergenova et al. 2003). Using SPARCS data, we demonstrated statistically significant elevation of hospitalization rates for respiratory infectious diseases, especially diseases such as chronic bronchitis (Kudyakov et al. 2004). The fact that we have demonstrated relationships between residence near waste sites and various diseases using two independent registries adds support to the hypothesis that living near such sites constitutes a risk of exposure and disease.

The observations reported in this investigation raise two important questions: What is the mechanism(s) involved, and what is the route(s) of exposure? Exposure to PCBs and dioxins is known to increase atherogenic serum lipid levels in both animals (Lind et al. 2004; Lovati et al. 1984) and humans (Calvert et al. 1996; Chase et al. 1982; Hu et al. 2003). Most likely, these actions are secondary to gene and enzyme induction in the liver resulting from exposure to POPs, which are difficult to metabolize (Boll et al. 1998). In addition, these contaminants cause direct damage to endothelial cells via oxidative stress (Choi et al. 2003; Hennig et al. 2002; Slim et al. 1999). The combination of an elevation in serum lipids with damage to endothelial cells would be expected to increase the risk of cardiovascular disease.

With regard to the route of exposure, the present and our previous observations (Baibergenova et al. 2003; Carpenter et al. 2001, 2003; Kudyakov et al. 2004) are most consistent with air transport of contaminates, both in vapor phase and particulate bound, being the major route of spread, with inhalation of these semivolatile compounds a major (and underappreciated) route of exposure. Ingestion is usually considered to be the most important route of exposure to POPs, but what people eat is not defined by the ZIP code in which they live. Consumption of contaminated fish is an important route of exposure to PCBs and dioxins, but even persons who engage in sports fishing are not defined by ZIP code of residence. There is increasing evidence that although PCBs and other POPs are not as volatile as some other organic pollutants, they are present in air at high levels around contaminated sites (Hermanson et al. 2004), and they can be absorbed from air and cause biologic effects in animals (Casey et al. 1999; Imsilp et al. 2005). POPs also bind to particulates, which can spread to nearby residences by air currents and can be either breathed in or unintentionally ingested. Although exposure to some of the contaminants at other-waste sites, especially some metals, is also associated with an increased risk of CHD, the fact that these compounds are not very volatile may explain why we did not find a statistically significant relationship between residence in ZIP codes containing these contaminants and CHD.

Several important confounders could explain these observations, particularly SES and behavioral risk factors. Harmful behavioral patterns and unfavorable environmental exposures associated with development of diseases have higher prevalence among lower social classes. Woodward and Boffetta (1997) report differences in exposure to environmental factors among different social classes. People of lower SES are more likely than those of higher SES to reside near polluted sites. New sources of pollution are likely to be placed in poorer neighborhoods, and populations of low SES tend to migrate to such disadvantaged areas for economic reasons (Woodward and Boffetta 1997). However, our results are adjusted for the median household income. Income is a commonly used proxy measure of SES (Berkman and Macintyre 1997; Krieger et al. 1997; Moss and Krieger 1995). Thus, an unequal distribution of socially disadvantaged population as a possible explanation of elevation in hospitalization rates for CHD in residents of POP ZIP codes is unlikely. Our results also control for age, race, and type of health insurance. In the subset of POP sites along the Hudson River, the average family income is higher than in the rest of New York State, yet rates of hospitalization for CHD and AMI are even more elevated than in the rest of the POP sites.

Health insurance coverage is also related to SES (Blackstock et al. 2002; Bradley et al. 2002; Rogers et al. 2000; Schneider et al. 2002). The effect of health insurance on mortality (lacking insurance is associated with higher mortality) is comparable with effects of education and income (Franks et al. 1993). For CHD, patients covered by the most comprehensive types of insurance (e.g., Blue Cross) and Medicare had lower rates than did those covered by Medicaid or not insured (self-pay). The opposite was observed for AMI patients (except for the subgroup of the Hudson River area residents): Those covered by the most comprehensive types of insurance and Medicare had higher rates. This probably reflects the fact that the CHD and AMI populations are different, with CHD being more heterogeneous and including AMI. Many of the chronic forms of CHD are not emergencies, and patients with good insurance are more likely to seek help and be diagnosed in the early stages of the disease. As a result, their chances for progression of CHD to more severe forms, such as AMI, are lower. In contrast, those covered by Medicaid or not insured are less likely to seek health care for conditions that are not emergencies. As a result, they present at hospitals with AMI.

Fine particulate air pollution is another well-documented risk factor for cardiovascular disease (Laden et al. 2000). Although there is no information on particulate levels in every ZIP code, the information available through the network of air monitoring stations in New York does not indicate that fine particulate levels explain the results obtained. However, additional study of air pollution as a possible confounder is warranted.

Our study is not free from limitations above and beyond the usual limitations of ecologic investigations (Morgenstern 1982). The SPARCS database contains information on discharges from state-regulated hospitals only. Data from federally regulated hospitals, including those operated under auspices of the Veterans Administration, are not available from the SPARCS database. Also, there is no information in SPARCS on how many times a patient was hospitalized, which prevents us from drawing conclusions on CHD incidence. Measurement of CHD incidence would provide stronger support of a cause–effect relationship between exposure to the pollutants and CHD development than only rates of CHD diagnosis on hospitalization. This is somewhat less of a problem with AMI, because repeated hospitalizations of the same person (i.e., hospitalizations for subsequent myocardial infarction) do not happen often. So to some degree AMI incidence can be approximated by hospitalization rate. A major limitation is that ZIP code of residence is a very crude measure of exposure. ZIP codes are of varying size, and we have not controlled for the location of the waste site within the ZIP code, because we have only the ZIP code of patient residence, not street address. However, if anything, these limitations would be expected to result in an underestimation of the true relationship. Although we have to some extent controlled for behavioral confounders through use of BRFSS data and air pollution through use of data from the network of monitoring stations in New York, neither of these data sets has information at the level of every ZIP code.

In summary, we determined that residency in POP-contaminated sites is associated with increased rates of hospitalization for CHD and AMI. Although the cross-sectional design of the study prevents us from making definitive conclusions on causal inference, the results support the hypothesis that exposure to PCBs, dioxins/furans, and/or persistent pesticides as a result of living near a hazardous waste site results in an elevated risk of CHD.

## Figures and Tables

**Table 3:** Hospital discharge rates for AMI in New York (other than New York City) as a function of residence near hazardous waste sites, sex, age, income, types of health insurance, and race.

Parameter	Variable name	OR (95% CI)
Contamination (vs. clean site)		
POPs	POP	1.200 (1.034–1.393)
Other waste	OTH	1.074 (0.927–1.244)
Sex (vs. female)	MALE	2.014 (1.781–2.279)
Age [years (vs. 25–34)]	AGE	
35–44		5.560 (4.347–7.111)
45–54		16.794 (13.237–21.306)
55–64		37.375 (29.424–47.480)
65–74		59.710 (45.943–77.603)
≥ 75		104.199 (79.464–133.647)
Median household income range [quartiles (vs. < $30,312.50)]	INCOME	
$30,312.50–35,714.50		0.940 (0.791–1.116)
$35,715–47,348.50		0.900 (0.759–1.068)
> $47,349		0.673 (0.568–0.797)
Health insurance coverage (vs. Medicaid/self-pay)		
Most comprehensive types of health insurance (Blue Cross, worker’s compensation, other government, insurance company, HMO, no-fault, self-insured)	BLUEX	1.680 (1.440–1.961)
Medicare	MCARE	2.058 (1.716–2.469)
Race (vs. Asian/Pacific Islander)		
Caucasian	CAU	1.372 (1.149–1.639)
African American	AA	1.538 (1.283–1.843)
Native American	NA	1.620 (1.307–2.008)

Abbreviations: CI, confidence interval; HMO, health maintenance organization; OR, odds ratio.

**Table 2:** Hospital discharge rates for CHD in New York (other than New York City) as a function of residence near hazardous waste sites, sex, age, income, type of health insurance, and race.

Parameter	Variable name	OR (95% CI)
Contamination (vs. clean site)		
POPs	POP	1.150 (1.029–1.286)
Other waste	OTH	1.041 (0.919–1.180)
Male sex	MALE	1.726 (1.573–1.893)
Age [years (vs. 25–34)]	AGE	
35–44		6.510 (5.422–7.818)
45–54		28.786 (24.059–34.446)
55–64		81.191 (76.836–97.164)
65–74		180.098 (148.725–218.066)
≥ 75		301.992 (249.236–365.951)
Median household income range [quartiles (vs. < $30,312.50)]	INCOME	
$30,312.50–35,714.50		0.915 (0.802–1.043)
$35,715–47,348.50		0.831 (0.729–0.947)
>$47,349		0.658 (0.577–0.750)
Health insurance coverage (vs. Medicaid/self-pay)		
Most comprehensive types of health insurance (Blue Cross, worker’s compensation, other government, insurance company, HMO, no-fault, self-insured)	BLUEX	0.525 (0.467–0.590)
Medicare	MCARE	0.602 (0.532–0.682)
Race (vs. Asian/Pacific Islander)		
Caucasian	CAU	1.713 (1.503–1.952)
African American	AA	1.992 (1.741–2.279)
Native American	NA	1.556 (1.339–1.808)

Abbreviations: CI, confidence interval; HMO, health maintenance organization; OR, odds ratio.

**Table 1:** Sociodemographic characteristics of the study population by categories of ZIP codes of residence: number (%) of person-year hospitalizations for 993–2000.

Characteristic	POP	Other waste	Clean
Total population (*n* = 56,078,068)	14,385,988 (25.65)	16,093,148 (28.70)	25,598,932 (45.65)
Sex			
Male	6,740,660 (46.86)	7,586,200 (47.14)	12,190,288 (47.62)
Female	7,645,328 (53.14)	8,506,948 (52.86)	13,408,644 (52.38)
Age (years)			
25–34	3,268,864 (22.72)	3,557,760 (22.11)	5,479,460 (21.41)
35–44	3,413,468 (23.73)	3,830,688 (23.80)	6,162,816 (24.07)
45–54	2,595,680 (18.04)	3,102,428 (19.28)	5,130,716 (20.04)
55–64	1,932,692 (13.43)	2,262,196 (14.06)	3,596,760 (14.05)
65–74	1,716,204 (11.93)	1,828,000 (11.36)	2,860,344 (11.17)
≥ 75	1,459,080 (10.14)	1,512,076 (9.40)	2,368,836 (9.24)
Race			
Caucasian	12,889,508 (89.60)	14,402,696 (89.50)	23,591,292 (92.16)
African American	1,155,728 (8.03)	1,303,800 (8.10)	1,375,224 (5.37)
Native American	56,552 (0.39)	43,616 (0.27)	72,124 (0.28)
Asian/Pacific Islander	284,200 (1.98)	343,036 (2.13)	560,292 (2.19)
Median household income range (quartiles)			
< $30,312.50	3,021,896 (21.01)	2,313,260 (14.37)	3,038,588 (11.87)
$30,312.50–35,714.50	3,136,328 (21.80)	2,301,088 (14.30)	3,547,376 (13.86)
$35,715–47,348.50	4,340,476 (30.17)	3,413,996 (21.21)	4,693,148 (18.33)
>$47,349	3,887,288 (27.02)	8,064,804 (50.11)	14,319,820 (55.94)

**Table 4:** Hospital discharge rates for CHD in a subset of POP sites along the Hudson River compared with clean ZIP codes in all New York except New York City.

Parameter	Variable name	OR (95% CI)
Contamination (vs. clean site)		
POPs	POP	1.358 (1.185–1.557)
Sex (vs. female)	MALE	1.669 (1.500–1.857)
Age [years (vs. 25–34)]	AGE	
35–44		6.442 (5.216–7.955)
45–54		27.741 (22.560–34.110)
55–64		78.783 (64.065–96.883)
65–74		180.170 (144.373–224.820)
≥ 75		297.020 (237.817–370.962)
Median household income range [quartiles (vs. < $30,312.50)]	INCOME	
$30,312.50–35,714.50		0.882 (0.757–1.027)
$35,715–47,348.50		0.833 (0.714–0.971)
> $47,349		0.624 (0.535–0.727)
Health insurance coverage (vs. Medicaid/self-pay)		
Most comprehensive types of health insurance (Blue Cross, worker’s compensation, other government, insurance company, HMO, no-fault, self-insured)	BLUEX	0.542 (0.473–0.621)
Medicare	MCARE	0.618 (0.535–0.713)
Race (vs. Asian/Pacific Islander)		
Caucasian	CAU	1.566 (1.343–1.826)
African American	AA	1.873 (1.600–2.193)
Native American	NA	1.529 (1.282–1.824)

Abbreviations: CI, confidence interval; HMO, health maintenance organization; OR, odds ratio.

**Table 5:** Hospital discharge rates for AMI in a subset of POP ZIP codes along the Hudson River compared with clean and other-waste ZIP codes in all New York except New York City.

Parameter	Variable name	OR (95% CI)
Contamination (vs. clean site)		
POPs	POP	1.391 (1.185–1.632)
Sex (vs. female)	MALE	2.038 (1.802–2.306)
Age [years (vs. 25–34)]	AGE	
35–44		5.772 (4.491–7.418)
45–54		18.601 (14.603–23.696)
55–64		43.645 (34.257–55.606)
65–74		90.432 (69.860–117.062)
≥ 75		163.973 (125.952–213.471)
Median household income range [quartiles (vs. < $30,312.50)]	INCOME	
$30,312.50–35,714.50		0.960 (0.805–1.145)
$35,715–47,348.50		1.056 (0.882–1.264)
> $47,349		0.672 (0.565–0.799)
Health insurance coverage (vs. Medicaid/self-pay)		
Most comprehensive types of health insurance (Blue Cross, worker’s compensation, other government, insurance company, HMO, no-fault, self-insured)	BLUEX	0.602 (0.516–0.703)
Medicare	MCARE	0.585 (0.496–0.690)
Race (vs. Asian/Pacific Islander)		
Caucasian	CAU	1.269 (1.062–1.518)
African American	AA	1.259 (1.048–1.513)
Native American	NA	1.481 (1.173–1.869)

Abbreviations: CI, confidence interval; HMO, health maintenance organization; OR, odds ratio.
